# Bovine Colostrum Treatment of Specific Cancer Types: Current Evidence and Future Opportunities

**DOI:** 10.3390/molecules27248641

**Published:** 2022-12-07

**Authors:** Ahmad R. Alsayed, Luai Z. Hasoun, Heba A. Khader, Iman A. Basheti, Andi Dian Permana

**Affiliations:** 1 Department of Clinical Pharmacy and Therapeutics, Applied Science Private University, Amman 11931, Jordan; 2 Department of Clinical Pharmacy and Pharmacy Practice, Faculty of Pharmaceutical Sciences, The Hashemite University, P.O. Box 330127, Zarqa 13133, Jordan; 3 Faculty of Pharmacy, Hasanuddin University, Makassar 90131, Indonesia

**Keywords:** bovine colostrum, cancer, treatment, natural products, chemotherapy, anti-cancer, lactoferrin

## Abstract

Worldwide, the incidence of cancer is on the rise. Current cancer treatments include chemotherapy, radiation therapy, and surgery. Chemotherapy and radiation treatment are typically associated with severe adverse effects and a decline in patients’ quality of life. Anti-cancer substances derived from plants and animals need to be evaluated therapeutically as it is cost-effective, have fewer side effects, and can improve cancer patients’ quality of life. Recently, bovine colostrum (BC) has attracted the interest of numerous researchers investigating its anti-cancer potential in humans. Dressings loaded with BC are beneficial in treating chronic wounds and diabetic foot ulcers. Lactoferrin, a glycoprotein with potent anti-oxidant, anti-inflammatory, anti-cancer, and anti-microbial effects, is abundant in BC. The BC pills successfully promote the regression of low-grade cervical intraepithelial neoplasia when administered intravaginally. The biological, genetic, and molecular mechanisms driving BC remain to be determined. Oral BC supplements are generally well-tolerated, but some flatulence and nausea may happen. To evaluate the therapeutic effects, long-term safety, and appropriate dosages of BC drugs, well-designed clinical trials are necessary. The purpose of this article is to emphasize the anti-cancer potential of BC and its constituents.

## 1. Introduction

Cancer is a significant public health concern that requires an immediate response to be managed. According to a prediction report, there will be 27.5 million new cancer cases yearly by 2040. Compared to the recent statistics, these values represent an increase of 61.7% [1].

At this point, most cancer patients rely on standard anti-cancer treatments such as chemotherapy, radiation therapy, and surgery. In addition, numerous naturally occurring substances produced by plants have been indicated to have direct roles in the treatment and prevention of cancer [2,3,4].

Infants fed breast milk have a reduced incidence of gastrointestinal (GI) tract infections than those fed formula or cow’s milk [5]. GI disorders can result in slowed physical growth and neurodevelopment, impaired immunological function, nutrient malabsorption, and early vulnerability to other disorders such as allergies and asthma. Colostrum works as a broad-spectrum anti-bacterial agent that protects newborns from GI infections and contributes to physical development, immune function, and the formation of the GI tract. Colostrum promotes the repair of the GI system and protects adults from gut pathogens (bacteria, viruses, fungus, and yeast) and leaky gut syndrome. Firstly, milk or colostrum contains much more physiologically active peptides, anti-oxidants, anti-inflammatory compounds, and growth-stimulating chemicals than later milk [6,7]. According to previous evidence [8], a weakened immune system in newborns causes some GI disorders. Colostrum consumption provides the foundation for life-long immunity. In rare situations, a newborn’s immunity is weakened due to a colostrum shortage or breast-feeding problems [9]. As a result, GI disorders manifest during adolescence or adulthood due to an impaired immune system. Neonatal consumption of colostrum is essential for physical growth, appropriate immune system development, and prevention of GI diseases later in life.

Colostrum provides newborns with nutrition in a highly concentrated, low-volume form. Due to its laxative characteristics, colostrum facilitates the passage of the baby’s first feces and aids in the removal of excess bilirubin to prevent jaundice [7]. Jaundice, anemia, liver cirrhosis, and Gilbert’s syndrome can result from excessive bilirubin accumulation in neonates [10,11,12].

Evidence shows that bovine colostrum (BC) includes nearly ninety bioactive compounds. Immunoglobulins and growth factors, antibodies, amino acids, oligosaccharides, anti-bacterial compounds, and immunological regulators (such as lactoferrin) are all examples of these bioactive molecules [13]. Furthermore, BC is abundant in vitamins and minerals (Table 1).

According to research, BC is 100 to 1000 times more powerful than human colostrum. Thus, human newborns benefit from the BC-supplemented formula, which provides passive immunity and growth ingredients for physical and GI development. BC is a novel therapeutic nutraceutical for children and adults [5].

The central theme of this review is to address the potential benefits of BC components in children and adults for treating cancer.

## 2. Cancer Overview and Treatment

Cancer is one of the most significant causes of mortality and morbidity globally [19,20]. According to a previous report [21], roughly 14.1 million people have cancer, and approximately 9.6 million cancer-related fatalities were documented in 2018 worldwide. From a mortality standpoint, one of every six fatalities is cancer related [22]. The most prevalent kinds of cancer are carcinoma, sarcoma, leukemia, lymphoma, melanoma, lung, colorectal, prostate, and breast [23,24,25,26,27,28,29,30,31]. Stomach, colorectal, and lung cancer are prevalent in both sexes, whereas liver and prostate cancers are more prevalent in males, while breast and cervical cancer are more prevalent in women. Presently, gastric cancer is one of the world’s most serious diseases. According to global cancer statistics, stomach cancer ranks as the fourth most prevalent form of the disease worldwide [4]. The American Cancer Society predicts that 1,918,030 new cancer cases and 609,360 cancer-related deaths will occur in the United States by 2022 [32].

Cancer is a disease in which cells divide uncontrollably in a body component or organ, ending in a tumor or carcinoma. Genetic, epigenetic, and environmental variables in the onset and progression of cancer play important roles. Two types of tumors exist: benign or noninvasive and malignant or invasive. The malignant cancer cells can infect neighboring tissues or organs and even migrate to distant parts of the body via blood or the lymphatic system, resulting in forming a new tumor far from the initial site [33]. Cancer, a prevalent malignant process, is characterized by cells with biological characteristics such as cell differentiation, aberrant proliferation, uncontrolled development, invasion, and metastasis. Its occurrence is frequently a complex process involving several components and processes [33].

Current cancer treatment methods include chemotherapy, radiation, bone marrow transplants, and surgery. However, these therapies have drawbacks and limitations; for example, radiation therapy causes indirect damage to surrounding tissues, chemotherapy results in toxicity to vital organs and also causes drug resistance, and surgical interventions can sometimes lead to tumor recurrence [34,35,36]. In addition to targeted drug delivery and immunotoxin therapy, recent developments in cancer treatment are reported [36]. Immunotoxin is a conjugated protein that combines a toxin with a targeted conjugate. Through endocytosis, these immunotoxins invade cancer cells and cause cell death. Chemotherapy has detrimental effects on important organs when used to treat stomach cancer [37,38]. Therefore, developing less hazardous therapeutic drugs for the prevention and treatment of stomach cancer is urgently required [39].

More than 60% of the currently approved cancer drugs or drug candidates are derived from natural sources [33]. Moreover, the wide range of natural products provides lead compounds that can be changed structurally. Their discovery dramatically expands the number of anti-cancer compounds. Their ability to attack multiple targets and have low toxicity has made them more important in preventing and treating cancer. Accordingly, natural drug-based anti-cancer therapies might become the most promising due to the rising cost of Western medicine research and development and the protracted process.

Based on their biological properties and action targets, natural medicines can be categorized as pharmaceuticals with direct action or drugs with indirect action. The first group comprises medications that target tumor cells directly. Some natural products, such as camptothecin, act on topoisomerase targets and prevent DNA replication in tumor cells by inhibiting topoisomerase activity [40]; taxanes [41] inhibit microtubule formation by binding to microtubule-associated proteins [42]. Other indirect methods of cancer prevention include enhancing the tumor microenvironment, inhibiting tumor angiogenesis and cancer cell invasion or adhesion [43], reversing multidrug resistance in tumor cells [44], and activating immune cells to regulate the immune function of the body [45]. The 2018 Nobel Prize in physiology or medicine was awarded to James Allison’s team for pioneering the concept of “immune checkpoint” and demonstrating for the first time that the CTLA-4 antibody enhances immunosuppression of tumor development in mice, making immunotherapy a promising cancer treatment.

With the advancement of analytical methodologies and the advent of new analytical instruments, the quantity of fundamental research on natural products in cancer treatment has increased dramatically. In recent years, the quantity of relevant patent literature and scientific journals has also increased significantly. It is difficult to comprehend the evolution and current status of natural anti-cancer medications given the abundance of information available.

## 3. Bovine Colostrum

Colostrum, the first milk produced after birth, offers nutrition, protective, and trophic components for the newborn mammal, contributing to the development and integrity of the GI system and the infant’s immune response [46]. Colostrum is a thick, sticky, yellowish liquid that provides nutrition and immunity and protects against microbial infections [47]. It can neutralize toxins and exhibits anti-bacterial action, making it a possible dietary intervention for treating or preventing chemotherapy-induced oral and intratesticular toxicity [46,48,49,50]. Some impacts may be species-specific, while multiple species may share others [51,52]. Compared to formula milk, exclusive feeding with intact BC improves GI responses to chemotherapy in piglets, according to pre-clinical investigations [53,54,55]. In these pre-clinical models, BC reduced gut toxicity by retaining intestinal function in terms of food absorption capacity, enzyme activity at the brush border, intestinal barrier function, and decreasing tissue inflammatory cytokine concentrations.

The major bioactive components of BC and their functions in children and adults are summarized in Table 1. The BC acquired from cows, and buffaloes possess more immunoglobulins than human colostrum, and human infants could benefit by consuming BC [56]. BC is usually considered safe in humans, whereas some people may initially experience nausea and flatulence, which declines over time. In addition, BC should not be given to individuals allergic to milk and milk products.

## 4. Role of BC in the Treatment of Cancer

A limited number of clinical trials with BC have shown anti-cancer effects in different forms of cancer [16,17]. Table 1 illustrates the anti-cancer benefits of lactoferrin, proline-rich polypeptides, conjugated linolenic acid (CLA), and alpha-lactalbumin. Lactoferrin, IgA, insulin-like growth factor, growth hormone, and epidermal growth factor concentrations are significantly greater in human colostrum than in BC [6,18].

### 4.1. Role of Lactoferrin and Lactalbumin in Cancer Therapy

Lactoferrin is a glycoprotein with powerful anti-oxidative, anti-inflammatory, anti-cancer, and anti-bacterial effects. Lactoferrin increases T-helper-1/T-helper-2/anti-inflammatory cytokine immunological response and production. In children, lactoferrin has been reported to prevent stomach infections, necrotizing enterocolitis, and late-onset sepsis [57,58,59].

Lactoferrin is a highly effective immunological modulator, anti-cancer drug, and tissue regenerator. Additionally, it can prevent the synthesis of pro-inflammatory cytokines. Lactalbumin is found in whey and can significantly boost immunological response and glutathione synthesis. Lactoferrin and lactalbumin have been reported to trigger apoptosis in malignant cells [60]. Lactoferrin has been observed to increase caspase-1 and IL-18, diminishing intestinal metastatic foci. Also detected is cytotoxic T and natural killer (NK) cell apoptosis triggered by lactoferrin. In addition, lactoferrin suppresses the activation of carcinogens via the hepatic CYP1A2 enzyme [61]. Because it can pass through the blood-brain barrier, lactoferrin can be used to carry chemotherapeutic drugs. This is especially useful for treating brain tumors [62]. It suggests that lactoferrin and whey lactalbumin can be utilized to treat cancer in conjunction with chemotherapy and radiation. This strategy would improve the chemotherapeutic efficacy of medications and minimize chemo- and radiotherapy, resulting in fewer cancer patients experiencing adverse side effects.

### 4.2. In Vitro Anti-Cancer Effects of BC Components

In vitro cell culture studies are used in selected cancer cell lines as a promising tool to determine the anti-proliferative and cytotoxic effects of potential anti-cancer agents isolated from natural sources or synthesized in the laboratory. In vitro cell culture studies provide clues about the mechanism of action of anti-cancer agents toward cancer cells. Anti-cancer effects of lactoferrin were evaluated using an MTT assay. The addition of lactoferrin in the culture medium inhibited the growth of cancer cell lines (MDA-MB-231 and MCF-7) [63].

Two milligrams per milliliter of purified lactoferrin inhibited the proliferation of esophageal cancer cell lines (KYSE-30) and HEK cancer cell lines. After 62 h, adding 500 g/mL lactoferrin to the culture medium decreased the cell viability of KYSE-30 cancer cells by 80%. The normal HEK cell line exhibited no effect. Analysis by flow cytometry revealed that lactoferrin caused apoptosis in KYSE-30 human esophageal cancer cell lines [39]. Table 2 summarizes the findings of in vitro studies conducted to evaluate the anti-cancer properties of BC components (lactoferrin, liposomal bovine lactoferrin, bovine lactoperoxidase, lactoferrin nanoparticles, and CLA) on various cancer cell lines (such as gastric, esophageal, colorectal, liver, lung, prostate, breast, and ovarian).

### 4.3. In Vivo Anti-Cancer Effects of BC Components in Humans and Animal Models

In order to evaluate the safety, efficacy, and toxicity of anti-cancer drugs, it is necessary to conduct preclinical research on suitable animal models after obtaining information from in vitro studies. Numerous anti-cancer research involving BC supplements and key components have been conducted on rodents. Rats and mice have been treated with lactoferrin and CLA for colorectal, lung, and esophageal malignancies. In preclinical trials, a reduction in colon tumor burden and a downregulation of VEGF expression were reported (Table 3) [66,69]. Only a small number of clinical trials on a few patients have been conducted to understand the anti-cancer potential of BC components. Based on the promising anti-cancer benefits of CLA in preclinical models, 24 breast cancer patients participated in an open-label clinical investigation. CLA was administered orally at 7.5 g/day for 20 days. It has been discovered that CLA inhibits the expression of fatty acid synthase (FASN) and lipoprotein lipase (LPL). The decreased activity of these biomarker enzymes indicates breast tumor growth suppression [70]. Another clinical investigation revealed that CLA (3 g/day) might be advantageous for rectal cancer patients undergoing chemoradiation [71]. Table 4 displays the results of preclinical and clinical studies on the effects of Lactoferrin, liposomal lactoferrin, and CLA on various forms of cancer.

### 4.4. BC for Chemotherapy-Induced GI Toxicity in Acute Lymphoblastic Leukemia (ALL)

The most prevalent kind of juvenile cancer is ALL. Cure rates continue to rise, with the 5-year overall survival rate over 90% and the long-term survival rate for children exceeding 80% to 90% [74,75,76]. However, anti-leukemic treatment is exacerbated by several toxicities. During ALL therapy, 2–4% of patients die due to treatment-related complications, primarily due to therapy-induced immune suppression and infections. In addition, nearly all patients get severe infections and chemotherapy-induced mucositis [77,78]. Mucositis is commonly believed to primarily impact the oral cavity, although it can also affect the entire GI tract [79,80,81]. GI toxicity may impair vital GI functions, resulting in microbial translocation with inflammatory and infectious consequences [79,82,83,84,85,86]. Consequently, multiple studies have shown links between oral and intestinal toxicity generated by chemotherapy and unfavorable treatment outcomes, such as fever, severe infections, and overall death [78,82,87,88]. However, therapeutic and prevention strategies are now restricted to highly specialized illness scenarios and dental care procedures for youngsters [48,89,90].

In a recent randomized, double-blind, placebo-controlled clinical trial, the researchers examined the impact of BC supplementation on fever, infectious morbidity, and mucosal toxicity during ALL induction therapy [38]. They observed no statistically significant influence on the critical outcome, fever days. Similarly, there was no significant effect on other infectious morbidities, such as days with neutropenic fever, the requirement for intravenous antibiotics, or C-reactive protein (CRP) levels. Patients in the placebo group had substantially higher peak NCI-oral mucositis scores than patients receiving the BC product, indicating a putative protective effect of BC supplementation on the oral mucosa. In a previous trial, whey protein concentrates were administered to patients undergoing hematopoietic stem cell transplantation. There was no effect found on mucositis. However, subgroup analysis suggested positive benefits on the duration and severity of oral mucositis in patients who received the highest protein supplement doses [91]. A putative protective impact of milk bio-actives on the GI mucosa is suggested by mouse experiments in which injection of components from bovine milk or whey had positive effects against chemotherapy-induced gut damage [92,93]. Most of the proposed beneficial chemicals in milk are present in greater concentration in colostrum than in mature milk, and complete BC has been recommended as a potential treatment for chemotherapy-induced mucositis [46,49,94]. Previous studies indicated that colostrum has gut-protective effects in pigs, relative to formula, in connection to chemotherapy [53,54]. However, the positive effects of increasing colostrum dosages could not be proven in pigs treated with chemotherapy and fed a normal milk diet [95], showing that both the dosing regimen and the control diet may be significant. In pre-clinical research, BC has been shown to sustain intestinal barrier function, improve nutritional absorption, and minimize intestinal inflammation [53,54].

During chemotherapy, in a previous clinical trial, most patients suffered febrile neutropenia, of which fewer than fifty percent may signify an established infection in pediatric cancer patients [96,97]. However, the origin of fever is frequently obscure and may result from systemic inflammation produced by other harmful consequences of chemotherapy. Several investigations have revealed a temporal relationship between GI damage, systemic inflammation, and fever in the absence of a detectable infection [83,98,99,100]. This is consistent with the clinical study’s findings, which indicate that the peak of gastrointestinal toxicity correlates with the peak of circulating CRP levels [38]. Overall, these findings indicate that hazardous events in the GI tract may be linked to microbial translocation and systemic immune activation. Consequently, interventions with implications for mucosal barrier preservation and compounds related to the protection, homeostasis, and modulation of inflammatory responses of mucosal surfaces after chemotherapy may help ameliorate the effects of chemotherapy on the GI tract and other complications [48,82]. Although BC had a putative protective impact on the oral mucosa, there were no significant effects on fever, intramuscular infection, inflammatory response, or bloodstream infections. Thus, systemic effects of BC supplementation could not be demonstrated.

Oral mucositis incidences were equivalent to those found in past trials of anthracycline-based chemotherapy and hematological malignancies [81,101]. Daily self-reporting toxicity rating return rates were also satisfactory and comparable to earlier reports [102].

The mentioned clinical study was limited by the small sample size and concerns with supplement compliance. Although the predicted number of patients was considered in the computation of sample size, the incidence of fever was lower than anticipated. The study had sufficient power to detect a difference in the number of fever days [38]. During ALL induction therapy, the administration of prophylactic BC had no effect on fever, infectious morbidity, or inflammatory reactions in this trial. Nonetheless, these preliminary results, although not conclusive, showed that BC supplementation may lessen the peak severity of oral mucositis, motivating additional research on mucositis to confirm these findings and evaluate its mechanism of action.

Prior studies of nutrition interventions for children with chronic conditions rarely measured compliance, and research on adults demonstrates difficulty in maintaining oral nutrition intervention adherence [103,104]. The reasons for lower adherence to oral supplements may include taste changes caused by chemotherapy, nausea, swallowing pain, and palatability concerns. In nutrition trials, it is essential to optimize the flavor, content, and texture of supplemental items and provide parents and pediatric patients undergoing chemotherapy with the proper support and instructions [104].

### 4.5. The Immunomodulatory Effects of BC on Colorectal Cancer

There is a significant interest in the inflammatory micro- and macro-environments of primary and secondary metastatic tumors in patients with colorectal cancer. The tumor’s microenvironment makes it easier for cancer cells to travel to other organs and establish themselves there, such as the liver and the lungs. Colostrum polyvalent immunoglobulins administered orally promoted the expression of anti-inflammatory cytokines known as interleukin (IL)-10 and IL-13 in patient-derived peripheral blood mononuclear cells [5]. In these cells, the release of inflammatory cytokines such as IL-1, IL-6, interferon, tumor necrosis factor, and IL-12 was reduced by colostrum polyvalent immunoglobulins that were given and delivered orally [105]. When colostrum polyvalent immunoglobulins were administered orally, immune cells and cells arising from tumors were both more prone to apoptosis. Using vitamin D3 as a cofactor resulted in an even greater improvement in the anti-inflammatory effects. In addition to being administered orally, the colostrum-derived polyvalent immunoglobulins displayed beneficial ex vivo effects on inflammatory cytokine responses. They increased the apoptosis of immune cells taken from patients diagnosed with colorectal cancer. This study provides evidence for the use of polyvalent immunoglobulins derived from colostrum that is provided orally to patients with colorectal cancer to modify stage-dependent local and systemic inflammation in these patients [5]. However, there is not yet adequate evidence to suggest that BC affects the immune microenvironment of tumors; despite this, further inquiry into the topic is warranted.

### 4.6. Targeted Oral Delivery of Paclitaxel through Exosomes Derived from BC

Paclitaxel is an anti-cancer medicine frequently used in the management of lung cancer and other types of cancer at earlier and more advanced stages. Paclitaxel, derived from solvents, is an effective cancer treatment, but it is often not well tolerated and is associated with significant side effects [106]. Exosomes and other naturally occurring nanocarriers are attracting much interest as a potential way to get around these limitations. According to a recent study, a tumor-targeted oral formulation of paclitaxel employing BC-derived exosomes not only enhances the clinical efficacy against orthotopic lung cancer but also reduces or eliminates the systemic and immunotoxicity of the traditional intravenous dose [106]. This finding was made possible by using exosomes derived from breast cancer cells. These findings will advance the exosome-mediated targeted oral delivery of paclitaxel as a therapeutic alternative to existing therapies by using the benefits of BC exosomes.

## 5. Future Opportunities

In this review, we summarized the nutritional health benefits of BC and the therapeutic potential and efficacy of commercially available BC powder, capsules, and tablets for treating a variety of malignancies. BC is a new nutraceutical, and innovative therapeutic products for children and adults are being developed. Future BC products could be a godsend in delivering non-hazardous, cost-effective, and economical alternative sources of natural cancer treatments. Nonetheless, there are a number of obstacles and possibilities that must be addressed. For example, well-designed, placebo-controlled, randomized clinical trials are required to determine BC supplements’ long-term safety, efficacy, and appropriate dosages. Other characteristics of BC nutraceuticals include the standardization of advantages derived from various cow and buffalo breeds. Moreover, proper manufacturing standards and standardized techniques are needed to make BC formulations. This is because synthetic pharmaceuticals and microbiological contaminants can be added to BC supplements. A more fundamental study is required to comprehend the mechanism of action of distinct anti-cancer components of BC.

Nutraceuticals are often comprised of materials separated or purified from vegetables and fruits, colostrum supplements, and dairy products and are marketed as non-pharmaceutical, cost-effective, and economical alternative cancer cures. Healthcare professionals and prominent specialists in medicine sometimes overlook the influence of nutraceuticals, functional foods, natural health products, dietary supplements, and probiotics. Nutraceuticals may be one of the most important factors in healing the global epidemic of chronic non-communicable diseases, such as obesity, diabetes, cardiovascular disease, and certain malignancies. However, the evidence-based dietary recommendations are plagued by poor quality science, a limited number of randomized, placebo-controlled studies, and unresolved disputes on the role of nutraceuticals in treating non-communicable diseases. For the production of BC nutraceuticals, good manufacturing practices and high-quality control requirements should be implemented. The tolerability of BC supplements and bioactive components should be subjected to rigorous post-marketing observation. As much as feasible, dairy farmers should be urged to collect BC in sterile and hygienic settings.

The BC is significantly more abundant in physiologically active peptides, anti-oxidants, anti-inflammatory compounds, and growth-stimulating substances than in mature milk. The benefits of BC for the health and disease of children and adults are widely documented. The hope is that BC supplements will significantly contribute to treating several types of cancer, diabetes, cardiovascular disease, necrotizing enterocolitis, inflammatory bowel disease or Crohn’s disease, and autoimmune disorders. Understanding the biological functions of various BC components is a significant task for nutritionists and dietitians, fundamental scientists, and medics.

Additionally, BC is effective against many bacterial, viral, fungal, and parasitic illnesses. Dressings containing BC are hypoallergenic, safe, and enhance wound healing. These dressings can be used as a supplement to treat severe wounds and burns. BC is higher in immunoglobulins than human colostrum and can be administered alongside conventional treatments to treat immunodeficiency illnesses and infections [6,7]. BC possesses intense anti-microbial activity, and it was recently reported that BC could alter the respiratory microbiome in a beneficial way [47]. In addition, BC may have anti-viral properties against the COVID-19 virus [107,108]. Lactoferrin is renowned for its anti-inflammatory and anti-bacterial effects. This potential notion may merit investigation.

## 6. Conclusions

The BC supplements have demonstrated efficacy in managing many human cancer cell lines (e.g., esophageal, colorectal, lung, breast, and ovarian cancer). Specific components of BC, including lactoferrin, CLA, and alpha-lactalbumin, are effective in the treatment of certain forms of cancer.

BC has shown anti-tumor properties in a limited number of studies conducted in vitro and in animals. Several components of BC have been shown to induce apoptosis in cancer cells and limit tumor growth. In addition, after being exposed to BC, NK cells are inhibited. However, people who are allergic to dairy products risk experiencing undesirable side effects, even though BC products are generally well accepted. In most cases, taking BC supplements for treating various cancers is very safe. The clinical interactions of BC, if any, with orally administered prescription or over-the-counter medications need to be investigated regarding bioavailability and pharmacokinetics. Additionally, the risk of such interactions needs to be monitored in patients using synthetic pharmaceuticals to treat co-morbid diseases.

## Figures and Tables

**Table 1:** Bovine colostrum (BC) components: functions and anti-cancer effects in children and adults.

Components	Function	Anti-Cancer Effect	BC (mg/mL)	Reference
Vitamins (A, B1, B2, B6, B12, D, E)	Promote growth and health			[14]
Minerals (Na, K, Ca, P, S, Mg, Mn, Zn, Cu, Fe)	Promote growth and health			[14]
Amino acids	Promote growth and health			[14]
Essential fatty acids	Promote growth and health			[14]
Immune factors Proline-rich polypeptide (PR)	Regulate function of the thymus gland, and reduce oxidative stress			[15]
Proline-rich polypeptides, conjugated linolenic acid (CLA)	Enhance T cell and natural killer (NK) cell activation	Ovaria, breast, rectal		[16,17]
Immunoglobulins				[15]
IgG	Neutralizes toxins and microbes		47.6	[6,18]
IgM	Destroy bacteria and highly anti-viral		4.2	[6,18]
IgA	Destroy bacteria and highly anti-viral		3.9	[6,18]
IgD, IgE	Destroy bacteria and highly anti-viral			[15]
Lactoferrin	Has anti-inflammatory, anti-bacterial, and anti-viral iron-binding glycoprotein with potential therapeutic use in cancer	Gastric, lung, colorectal, breast	100	[15,16,17]
Growth factors	Stimulate DNA synthesis, and promote the growth of cell and tissue			[15]
Epidermal growth factor			30–50 μg/L	[6,18]
Growth hormone	Stimulate DNA synthesis, and promote the growth of cell and tissue		<0.03 ng/L	[6,15,18]
Platelet-derived growth factor	Stimulate DNA synthesis, and promote the growth of cell and tissue			[15]
Insulin-like growth factor			10	[6,18]
Fat	Stimulate DNA synthesis, and promote the growth of cell and tissue Fat content in BC (6.7%) is higher than in human colostrum (3–5%).			[6,7,15]
Protein	Stimulate DNA synthesis, and promote the growth of cell and tissue Protein content in BC (14.9%) is significantly higher than human colostrum (0.8–0.9%).			[6,7,15]
Lactose	Stimulate DNA synthesis, and promote the growth of cell and tissue Lactose content in BC (2.5%) is significantly lower than human colostrum (6.9–7.2%).			[6,7,15]
Alpha-lactalbumin	Anti-viral, anti-tumor	Breast		[16,17]

**Table 2:** Anti-cancer effects of BC constituents on various cancer cell lines.

BC Component	Cancer Type	Dose	Result	Reference
Lactoferrin	Gastric cancer (AGS human stomach carcinoma cell)	500 μg/mL	80% cytotoxicity in AGS cell line	[64]
Lactoferrin	Human esophagus cancer cell (KYSE-30 esophageal squamous cell carcinoma)	500 μg/mL	After 20 and 62 h, the growth of azoxymethane (AOM)-induced aberrant crypt foci (ACF) was inhibited by 53% and 80%, respectively.	[39]
Lactoferrin	Lung cancer (human lung cancer cell line, A549)	0.9375–15 mg/mL	Lactoferrin lung cancer (human lung cancer cell line, A549) 0.9375 to 0.1 mg/mL Concentration-dependent suppression of VEGF mRNA and VEGF protein expression.	[65]
Liposomal bovine lactoferrin	Colorectal cancer (RKO and RCN-9 human CRC cells)	≥10 μg/mL	Significant (*p* < 0.01) inhibition of colon aberrant crypt foci growth occurred in the RKO and RCN-9 cells.	[66]
Bovine lactoperoxidase (LPO) and lactoferrin (LF) nanoparticles	Colorectal cancer (Caco-2), liver cancer (HepG-2), breast cancer (MCF-7), and prostate cancer (PC-3).	315–1388 μg/mL	NF-kB expression was inhibited by a factor of ten in Caco-2, HepG-2, and MCF-7 cells; NF-B mRNA levels were reduced by four in PC-3 cells, and Bcl-2 levels were reduced by a factor of fifteen in comparison to 5-fluorouracil treatment.	[67]
CLA	Ovarian cancer cells (SKOV-3 and A2780 cells)	7 μM CLA for 48 to 72 h	Reduction in E2F induces a ninefold increase in autophagolysosomes and a G1 cell cycle arrest in SKOV-3 and A2780 cell lines.	[65]
	Breast cancer cell line (MCF-7), colon cancer cell line (HT-29), (mouse fibroblast cell line Balb/3T3)	0.1–100 µg/mL	Reduced anti-apoptotic Bcl-2 expression	[68]

**Table 3:** Effects of BC components on some cancer types in humans and animals, both pre-clinically and clinically.

BC Components	Cancer Type	Dose	Results of the Pre-Clinical/Clinical Study	Reference
Lactoferrin	Lung cancer (12 transgenic mice)	300 mg/kg, 3 times a week	The expression of hVEGF-A_165_ was significantly reduced and suppressed tumor formation.	[69]
Liposomal lactoferrin	Colorectal cancer (thirty-six F344 male rats, 5-weeks-old, were used in the experiment)	1000 mg/kg/day in drinking water	Around a 43% decrease was observed in the colon tumor.	[66]
CLA	Breast cancer (24 women)	7.5 g/day in the form of capsules for 20 days	The decrease in FAS and LPL enzymes provides fatty acids for breast tumor growth.	[70,72,73]
	Rectal cancer (33 human volunteers)	3 g/day in the form of capsules for six weeks.	Significant changes occurred in TNF-α (*p* = 0.04), hs-CRP (*p* = 0.030), and MMP-9 (*p* = 0.040)	[71]

**Table 4:** The role of BC in prevention of tumor initiation, proliferation, and progression.

Role of BC	Anti-Cancer Effect of BC
Antagonizes the effect of a potent DNA methylating agent dimethylhydrazine	Prohibits tumor angiogenesis
Blocks the NF-kB pathway	Stops tumor formation and proliferation and induces apoptosis
Inhibits the formation of the carcinogen azoxymethane	Prevents the formation of aberrant crypt foci and subsequently reduces the number of viable colon precancerous cells.
Halts E2F2 transcription factor expression	Promotes cell cycle arrest and tumor formation
Conjugated linolenic acid in bovine colostrum inhibits fatty acid synthetase and lipoprotein lipase expression	Suppresses fatty acid synthesis and impairs breast cancer growth
Decreases the levels of MMP-2/9 enzymes	Inhibits tumor progression
Inhibits hVEGF expression	Hinders tumor angiogenesis (ex. Bovine lactoferrin inhibits lung cancer growth)

NF-kB, nuclear factor kappa B; hVEGF, human vascular endothelial growth factor; MMP-2/9, matrix metalloproteinase-2/9.

## Data Availability

Not applicable.
